# Pharmacy in France

**Published:** 1842-05

**Authors:** 


*Pharmacy in France.*—The School of Pharmacy in Paris comprises five titular professors, "*professeurs titulaires*," and three assistant professors, "*professeurs adjoints*." The other schools have three titular and two assistant professors. In each school there are also associated assistants, "*agrégés*," appointed for five years, who take the place of the professors in case of their absence, and assist at examinations. In the school in Paris there are five associate assistants, and three in the schools of Montpelier and Strasbourg. The titular and assistant professors are appointed by the minister of public instruction, from a double list of presentations, made, the one by the *School of Pharmacy*, and the other by the *Faculty of Medicine* of the town in which the school is situated. Each list of presentations contains the names of two candidates, but the same candidates may be presented both by the School of Pharmacy and by the Faculty of Medicine. No one can be named as titular professor who is not a doctor in physical sciences, and thirty years of age. The assistant professors are required to be licentiates in physical sciences, and twenty-five years of age. Both are required to have been admitted Pharmaciens in one of the schools of Pharmacy. The associated assistants are to be appointed by "*concours*," in a manner to be hereafter arranged in the council of public instruction. To be admitted to the "*concours*," it will be sufficient to produce the diploma of a Pharmacien and of a bachelor in physical sciences. The director of the school is to be chosen by the minister of public instruction, from among the titular professors. He is to be in office for five years, and is eligible for re-election. Each school is provided with a responsible secretary, chosen by the minister of public instruction, from among the titular or assistant professors. There are also one or more "*preparateurs*," who must have the degree of bachelor of physical sciences, and are appointed by the director, with the concurrence of the professors. The director appoints the officers and servants. The *instruction* in each school comprises:—

*First Year.*—Physics, Chemistry, and the Natural History of Medicines.

*Second Year.*—Natural History of Medicines, Materia Medica, and Pharmacy, properly so called.

*Third Year.*—Toxicology; and in the practical school, Chemical and Pharmaceutical manipulations.

No candidate can be admitted to an examination for the title of Pharmacien, who has not obtained the degree of bachelor of letters. Besides the two professors in medicine who are appointed to officiate at the examinations, three members of the College of Pharmacy must also be present, namely, two titular or assistant professors, and one associated assistant. The students of the Schools of Pharmacy, who have gained prizes at the "*concours*," are exempted from the fees. The amount remitted for each prize is to be regulated by the university. The names of the successful students are published.

The *receipts* and *expenditure* of the Schools of Pharmacy are carried to the national budget of public instruction. The titular Professor in Paris, is to receive a fixed annual salary of 4000 francs; in the departments of 3000 francs. The assistant Professors, in Paris, are to receive an annual salary of 2,400 francs; in the departments, 1,500 francs. The Director is to receive in addition, as a jointure, an annual stipend of 1,500 francs, in Paris, and 1000 francs in other colleges. The salary of the Secretary, in Paris, is 3000 francs; in the other schools, 1,500 francs. The salary of the Preparateurs, is 1,200 francs. The payment for attendance at the examinations is 10 francs for those functionaries who are called upon to officiate. The same is allowed to the professors, who are charged with the examination of herbalists. The fee for the annual certificate, granted to each student, is fixed at 36 francs in each of the schools. The charge for examinations remains unaltered; for the first examination, 200 francs; for the second, 200 francs; for the third, 500 francs. The expenses of operations and demonstrations, incurred during the third year, which are defrayed by the candidates, are fixed at 200 francs, in Paris, and 150 francs in the other schools.

The acquirement of the diploma of Bachelor of Letters, will not be required in the candidates for examination, until the 1st of February, 1844.—*Pharmaceut. Transactions, Oct.* 1, 1841.

